# Codon optimality modulates protein output by tuning translation initiation

**DOI:** 10.1101/2023.11.27.568910

**Published:** 2023-11-28

**Authors:** Elijah F Lyons, Lou C Devanneaux, Ryan Y Muller, Anna V Freitas, Zuriah A Meacham, Maria V McSharry, Van N Trinh, Anna J Rogers, Nicholas T Ingolia, Liana F Lareau

**Affiliations:** 1 Department of Molecular and Cell Biology,; 2 California Institute for Quantitative Biosciences,; 3 Department of Bioengineering, University of California, Berkeley

## Abstract

The impact of synonymous codon choice on protein output has important implications for understanding endogenous gene expression and design of synthetic mRNAs. Previously, we used a neural network model to design a series of synonymous fluorescent reporters whose protein output in yeast spanned a seven-fold range corresponding to their predicted translation speed. Here, we show that this effect is not due primarily to the established impact of slow elongation on mRNA stability, but rather, that an active mechanism further decreases the number of proteins made per mRNA. We combine simulations and careful experiments on fluorescent reporters to argue that translation initiation is limited on non-optimally encoded transcripts. Using a genome-wide CRISPRi screen to discover factors modulating the output from non-optimal transcripts, we identify a set of translation initiation factors including multiple subunits of eIF3 whose depletion restored protein output of a non-optimal reporter. Our results show that codon usage can directly limit protein production, across the full range of endogenous variability in codon usage, by limiting translation initiation.

## Introduction

The genetic code is elegant yet redundant, with 20 amino acids specified by 61 codons. The synonymous codon options are not exactly equivalent. Genomes have evolved to prefer some codons over others, and the differences between synonymous codons have real impact. As a ribosome moves along an mRNA, it spends more time decoding some codons than others, because some tRNAs are less available or interact less favorably with the translation machinery. High resolution methods have allowed a close look at translation, revealing surprising consequences for mRNA stability, protein output, and proper co-translational protein folding. Decoding the impact of synonymous codon usage is key to a more complete understanding of the output of the genome as well as better models for the design of synthetic mRNAs such as those used in vaccines.

It was not initially clear that total translation time would generally affect protein output. Older theoretical models had suggested that translation initiation (recently measured to take 30 seconds in an *in vitro* yeast system (Wang et al. 2022) was the rate-limiting step in protein synthesis, with elongation (6 amino acids per second in mammalian cells (Ingolia, Lareau, and Weissman 2011)) rarely able to limit output (Plotkin and Kudla 2011; Shah et al. 2013; Riba et al. 2019; Erdmann-Pham, Dao Duc, and Song 2020). However, groundbreaking results established solidly that codon choice does affect protein output across the full endogenous range. Most notably, slow translation can destabilize mRNAs and thereby reduce total protein production, a process termed codon optimality mediated decay (Presnyak et al. 2015; Bazzini et al. 2016; Narula et al. 2019; Q. Wu et al. 2019; Bae and Coller 2022). Studies show that the Not5 subunit of the CCR4-NOT deadenylation complex binds the empty ribosomal E-site when the A-site lacks an accommodated tRNA, promoting mRNA decay (Buschauer et al. 2020). This distinct ribosomal configuration is a hallmark of slow translation kinetics.

Variability in decoding time can be captured empirically with ribosome profiling, a high throughput method that captures the positions of individual ribosomes and shows their distribution along mRNAs (Ingolia et al. 2009). The experiment is more likely to sample ribosomes at positions that are decoded slowly, so the count of ribosome footprints at a position reflects the dwell time of a ribosome decoding that position, when considered relative to the average along that gene (Lareau et al. 2014). Thus, computational analysis of sequencing data can reveal the progression of ribosomes along transcripts with remarkable resolution.

**Figure 1:**
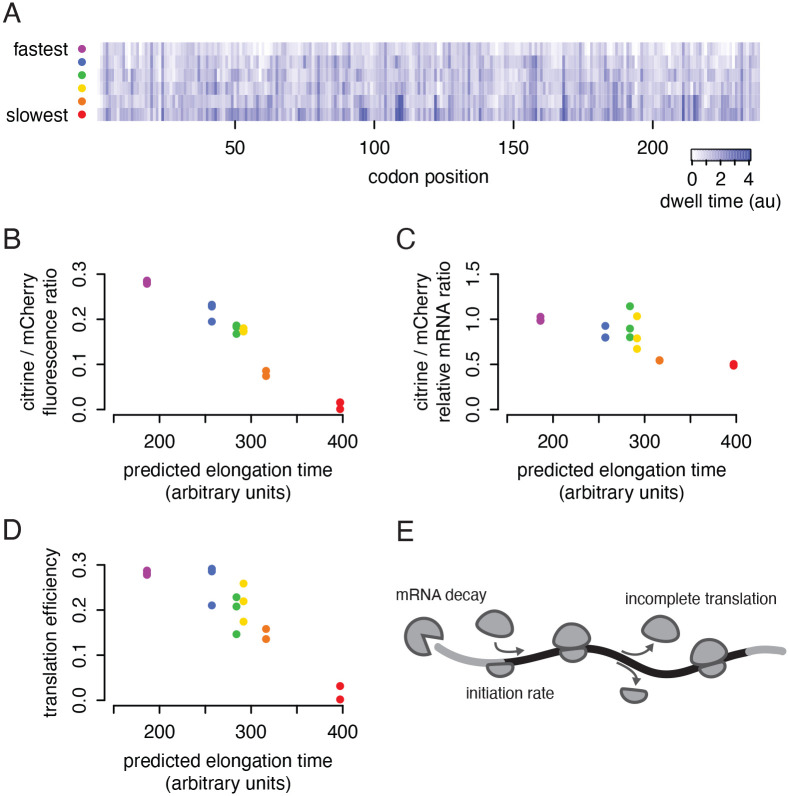
Non-optimal reporters produce less protein per transcript, beyond the effect of mRNA decay. a) Six synonymous citrine reporters depicted with their predicted ribosome dwell times at each position. Reporter sequences were designed in our previous work (Tunney et al, 2018), using a neural model that predicts the time the ribosome spends on each position of a coding sequence as a function of the identity of the codon being decoded in the ribosomal A site and the adjacent codons in the P and E sites and neighboring sequence. The reporters were designed to span the range of total decoding times from fastest to slowest possible. b) Flow cytometry measurements of citrine fluorescence normalized to mCherry fluorescence for each synonymous reporter. Median fluorescence ratio for 50000 yeast is shown for three isolates of each reporter. Data for panels b, c, and d are reanalyzed from Tunney et al, 2018. c) mRNA abundance of each synonymous citrine reporter normalized to mCherry mRNA abundance, determined by RT-qPCR. d) Translation efficiency of each synonymous reporter, calculated as the normalized fluorescence divided by normalized mRNA abundance. e) Schematic representation of factors that may limit translational output of non-optimal reporters. CDS shown in black, UTRs shown in gray.

In our previous work, we made use of ribosome profiling data to establish the connection between codon identity, ribosome speed, and protein output (Tunney et al. 2018). We used a neural network to learn the relationship between mRNA sequence and empirical ribosome footprint counts from the yeast *S. cerevisiae*, then predicted the total time to decode a particular mRNA and asked if this could predict protein output. To test this, we used our model to design six synonymous encodings of a fluorescent protein, citrine, with predicted decoding times spanning the range of endogenous yeast genes (Fig. 1A). When we expressed these reporters in yeast, we found a striking linear correspondence between the predicted decoding time and the protein output for each variant (Fig. 1B). The reporter encoded with the fastest codons produced seven-fold more fluorescence than the reporter encoded with the slowest codons.

In light of the new understanding of codon optimality mediated decay, we were surprised to find that the difference in output from our reporters was only partially explained by different mRNA abundances, with at most a two-fold difference in mRNA abundance between the fastest and slowest construct (Fig. 1C). The translation efficiency, or proteins produced per mRNA in a given amount of time, still tracked the predicted decoding time (Fig. 1D). This observation motivated the current study, in which we build on our previous work to identify the mechanisms by which slow codons limit protein output, beyond the effect of slow elongation on mRNA half-life. In a simple model where ribosomes initiate infrequently and elongation proceeds at a rate that is fast relative to initiation, and with no mechanisms sensing slow ribosomes, the total number of proteins made per mRNA in a given amount of time would not depend on the speed of elongation (Plotkin and Kudla 2011; Shah et al. 2013; Erdmann-Pham, Dao Duc, and Song 2020). Initiation would be rate-limiting and our constructs would each produce roughly the same protein output at steady state. An additional mechanism is required to account for a difference in translation efficiency.

Conceptually, the potential mechanisms fall into three categories. If elongation is slow relative to initiation, ribosome traffic jams could back up to the start codon and occlude it, preventing initiation until the jam is cleared (Plotkin and Kudla 2011; Shah et al. 2013; Chu et al. 2014; Tuller et al. 2010; Riba et al. 2019; Verma et al. 2019; Erdmann-Pham, Dao Duc, and Song 2020). This would not require any active mechanism beyond the physical process of ribosomes moving slowly along an mRNA. If elongation is not slow enough to cause this passive limitation, an active mechanism is implicated. For instance, abortive translation could prevent ribosomes from reaching the end of the coding region. Acute ribosome stalls and collisions in both yeast and mammalian cells generally result in decreased protein output via abortive termination coupled with mRNA degradation through no-go decay (NGD) or non-stop decay (NSD), followed by nascent chain degradation through ribosome-associated quality control (RQC), and similar mechanisms could affect our slow reporters (Shoemaker and Green 2012; Brandman and Hegde 2016; Meydan and Guydosh 2021; Brandman et al. 2012; Veltri et al. 2022). Finally, a feedback mechanism could sense slow elongation and limit initiation. A distinct pathway in which collided ribosomes inhibit translation initiation has recently been identified in mammalian cells, although this specific pathway is not thought to operate in yeast, which lack a homolog of a key component, 4EHP (Hickey et al. 2020; Juszkiewicz et al. 2020; Sinha et al. 2020). Recent work has demonstrated that slow elongation, even without collisions, can limit initiation to decrease protein output in human and fly cells (Barrington et al. 2022).

Here, we investigate how elongation is rate limiting in protein production in yeast across a wide range of codon optimality. We use reporter assays and a high-throughput CRISPR interference screen to disentangle the mechanisms linking slow elongation to lower protein output. Our results implicate a mechanism that limits initiation on transcripts with slow elongation. Our results also emphasize that the consequences of slow elongation depend substantially on the rate of translation initiation, with a tipping point between these two potentially rate limiting processes.

## Results

### Non-optimal reporters produce less protein per transcript, beyond the effect of mRNA decay

We first confirmed that the large difference in observed fluorescence between our synonymous citrine reporters was not due entirely to differences in mRNA abundance. Using the same six synonymous citrine sequences as in our earlier work (Fig 1A), we constructed yeast strains each containing one citrine reporter variant and a standard mCherry normalizing reporter. We confirmed that the protein output of the synonymous reporters closely tracked their predicted elongation time, with the fastest reporter producing 12 times more fluorescence than the slowest (Fig. S1A). We then measured the mRNA abundance of each citrine reporter with RT-qPCR. When normalized to mCherry mRNA, citrine mRNA abundance also closely tracked the predicted elongation time, but only across a narrower 2-fold range (Fig. S1B). Non-optimal reporter transcripts were indeed less abundant, in keeping with the well-observed relationship between slow codons and faster mRNA turnover (Presnyak et al. 2015), but mRNA abundance differences did not drive the majority of the difference in protein output. Dividing the total protein by total mRNA, we found that the translational efficiency (or proteins produced per mRNA in a given amount of time) still spanned a 6-fold range.

In this study, we will focus specifically on the output of our fastest and second-slowest reporters, which we refer to as ‘fast’ and ‘slow’ citrine, respectively. (The absolute slowest reporter falls well outside the range of codon usage of endogenous genes (Tunney et al. 2018).) The slow reporter produced only 65% of the mRNA of the fast reporter, and only 32% of the protein. Thus, each transcript of our slow reporter generated half as much functional mature protein per time as each transcript of our fast reporter (Fig. S1C).

### In a simple model, optimal and non-optimal reporters would have similar translation efficiency

**Figure 2:**
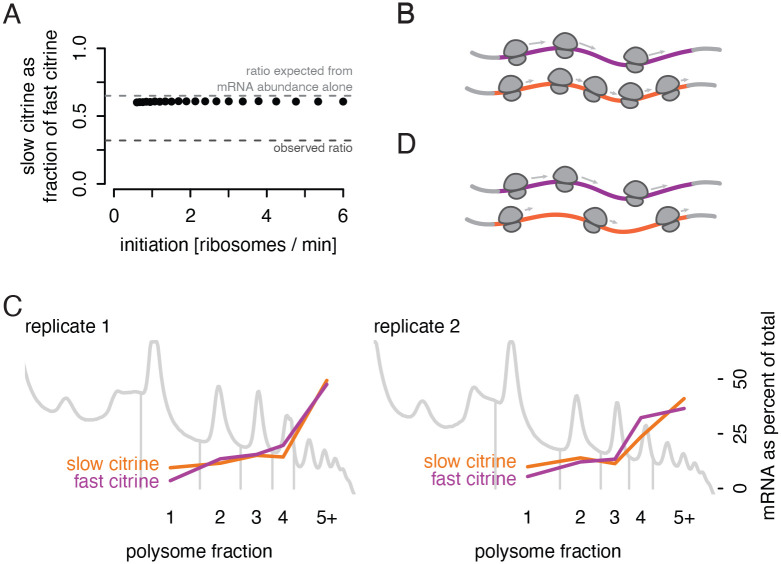
Measured translation output does not match predictions of a simple simulation. a) Simulated protein output from the TASEP model, shown as the ratio of slow reporter to fast reporter protein output, does not approach the observed ratio of protein output (dark gray dashed line) across a range of realistic initiation rates. Also shown is the protein output expected based only on mRNA differences (light gray dashed line). b) Schematic representation of expected ribosome density on fast (purple) and slow (orange) synonymous reporter transcripts if no additional regulation beyond mRNA decay occurs to limit translation of the slow reporter. c) Distribution of ribosome counts on fast and slow reporter mRNAs as determined by sucrose gradient fractionation of polysomes and RNA sequencing. To accurately compare reporter abundances in each fraction, a constant mass of human RNA was spiked into each fraction before RNA isolation. Abundance of each reporter in each fraction of the gradient is presented as a percentage of the total mRNA for that reporter. Fast and slow reporter strains were co-cultured to allow measurement from a single gradient; two replicates were performed with distinct isolates of the reporter strains. Polysome trace is depicted as 254 nm absorbance, gray. d) Alternative model explaining observed ribosome density on fast (purple) and slow (orange) synonymous reporter transcripts.

To confirm our understanding that differences in elongation speed on their own should not produce different amounts of protein, we implemented a simple stochastic TASEP (Totally Asymmetric Simple Exclusion Process) model (Fig. S2A) (MacDonald and Gibbs 1969; Zia, Dong, and Schmittmann 2011). Ribosome initiation rates and per-codon elongation rates were chosen to span a wide range of realistic values gathered from experimental data (Arava et al. 2003; Ingolia, Lareau, and Weissman 2011; Wang et al. 2022), and we simulated a shorter half-life for the slow reporter to match its observed mRNA abundance. In this simulation, with no additional feedback mechanisms beyond the impact of codon-optimality mediated decay, the ratio of protein abundance to mRNA abundance for the slow reporter was 92–93% that of the fast reporter (Fig. 2A; Fig. S2B). (This translation efficiency calculation factors in the decreased output due to the lower mRNA abundance\) This stood in contrast to our experiments in which the translational efficiency of the slow reporter was 50% that of the fastest reporter. Our results thus led us to consider additional mechanisms by which slow codons could affect protein output beyond the established effect of slow elongation on mRNA half-life. The scenarios where elongation could affect protein output have specific requirements: slow elongation must either cause fewer ribosomes to initiate or fewer ribosomes to terminate successfully or produce functional proteins.

### Lower than expected ribosome density on non-optimal transcripts implicates a mechanism limiting translation

To further test the possibility that some mechanism limits protein output from our slow reporters, we examined the ribosome density on our reporters with polysome profiling. In the absence of such a mechanism, a slow citrine mRNA would generally contain more ribosomes than a fast citrine mRNA (Fig. 2B). Equal initiation, but slower elongation, would lead to accumulation of ribosomes without affecting the total protein production per time.

To measure ribosome density empirically, we co-cultured yeast expressing our fast reporter and yeast expressing our slow reporter, used ultracentrifugation through a sucrose gradient to separate mRNAs by the number of associated ribosomes, then measured the abundance of citrine mRNA in each polysome fraction via RNA sequencing. We observed that fast and slow citrine mRNAs displayed very similar distribution across the polysome gradient fractions (Fig. 2C). Thus, the number of translating ribosomes on the slow reporter was notably decreased relative to the expected number (Fig. 2D). This result points to some mechanism reducing initiation on slow transcripts, removing ribosomes from slow transcripts (beyond the effects of codon optimality mediated decay), or destabilizing the final protein product.

### Known quality control pathways do not explain decreased protein output

**Figure 3:**
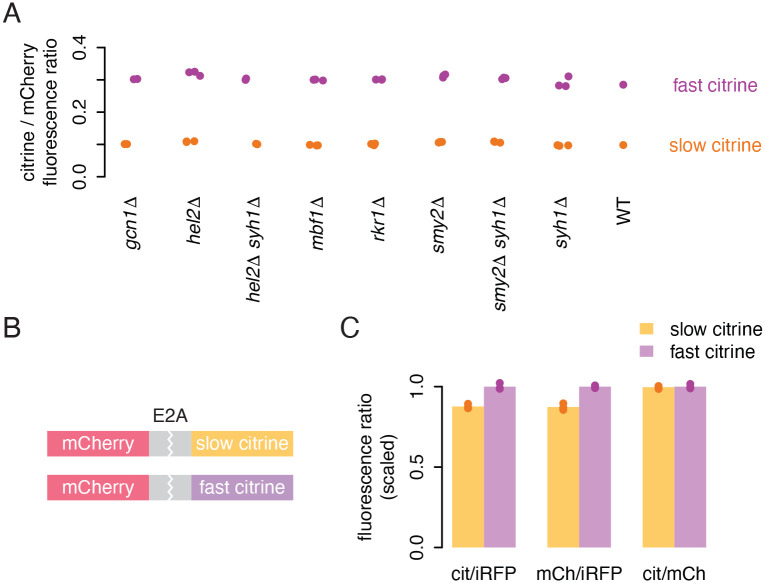
Lack of evidence for incomplete translation on non-optimal sequences. a) Flow cytometry measurements of fluorescence from fast and slow citrine reporters, as in figure 1B, in strains with deletions of genes implicated in ribosome associated quality control and related mechanisms. b) Schematic of dual fluorescence E2A constructs composed of an mCherry coding sequence with no stop codon, an E2A skipping sequence, and either the slow or fast citrine sequence. Yeast backgrounds also contain emiRFP for normalization. c) Flow cytometry measurements of citrine and mCherry fluorescence from fast and slow E2A constructs, each normalized to iRFP, as well as the ratio of citrine to mCherry. Citrine and mCherry measurements were each scaled to the average citrine or mCherry fluorescence, respectively, of the fast citrine reporter. The bar height shows the average of three isolates.

Aberrant translation has been shown to trigger regulation that can result in incomplete translation, so we next tested if known feedback pathways are active on our reporters. We deleted a number of key genes in these translational quality control pathways in yeast that expressed either the fast or slow citrine reporter along with a normalizing mCherry reporter. We deleted genes encoding factors that are known to associate with or act downstream of ribosome collisions, including the NGD/NSD ribosome collision detectors *HEL2* and *MBF1* (Sitron, Park, and Brandman 2017; Matsuo et al. 2017; Sinha et al. 2020), integrated stress response ribosome collision sensor *GCN1* (Sattlegger and Hinnebusch 2005; Pochopien et al. 2021), and E3 ubiquitin ligase *RKR1* (mammalian *LTN1*) which tags nascent chains for degradation in RQC (Bengtson and Joazeiro 2010). We also deleted *SYH1* and *SMY2* and made double knockouts of *SYH1* with both *SMY2* and *HEL2* to confirm the absence of regulation homologous to the mammalian GIGYF2–4EHP pathway in which collisions limit translation initiation (Hickey et al. 2020; Veltri et al. 2022). We observed no difference in normalized citrine fluorescence in any knockout strains compared to the original citrine strains (Fig. 3A), indicating that these quality control pathways are not actively reducing the protein output of our non-optimal reporters. The results suggest that our synonymous reporters, designed to capture the normal endogenous range of codon usage, are not subject to quality control mechanisms that respond to aberrant translation. Rather, an as-yet-uncharacterized mechanism may limit the number of ribosomes completing protein synthesis, either through repression of initiation or unsuccessful translation.

### Incomplete translation does not explain lower output of non-optimal sequences

To differentiate between these possibilities, we designed dual fluorescent reporter constructs with an upstream mCherry sequence followed by a viral E2A skipping element and either our fast or slow citrine variant (Fig. 3B). The E2A element causes ribosomes to skip catalysis of one peptide bond, releasing the upstream product while translation proceeds into the second sequence (Donnelly et al. 2001). Mechanisms by which slow elongation limits translation initiation or destabilizes the mRNA would reduce mCherry output to the same extent as citrine output from these E2A constructs. If, on the other hand, slow elongation caused some uncharacterized effect such as ribosome removal or destabilization of citrine protein, the effect would be decoupled, with less effect on mCherry output than citrine output.

We expressed the two E2A constructs in yeast along with iRFP as a normalizer and measured fluorescence via flow cytometry (Fig. 3C). As expected, the slow citrine E2A construct produced less citrine fluorescence than the fast citrine construct, although the difference was attenuated relative to our original reporters. (This could indicate that the skipping sequence itself creates a translation stall and collision, decreasing output of both the slow and fast reporters (C. C.-C. Wu et al. 2020).) Importantly, we observed that the slow citrine construct also produced less mCherry fluorescence, and that the ratio of citrine to mCherry fluorescence for both E2A constructs was identical. This indicates that the reduction in output caused by slow ribosomes on the downstream sequence is imposed equally on both the upstream and downstream sequences. This result is not consistent with the possibility of ribosome removal from the non-optimal citrine sequence on the slow E2A construct or a difference in stability of the protein products from fast vs slow citrine sequences. Instead, it provides further support for a combination of mRNA destabilization and impaired initiation limiting output from slowly translated sequences. In the absence of ribosome removal, there must be fewer ribosomes initiating on slow reporter transcripts to generate the similar polysome profiles of fast and slow reporter mRNAs. Given these results, we considered mechanisms that might limit initiation on our non-optimal reporters.

### Passive start codon occlusion does not explain lower output of non-optimal sequences

**Figure 4:**
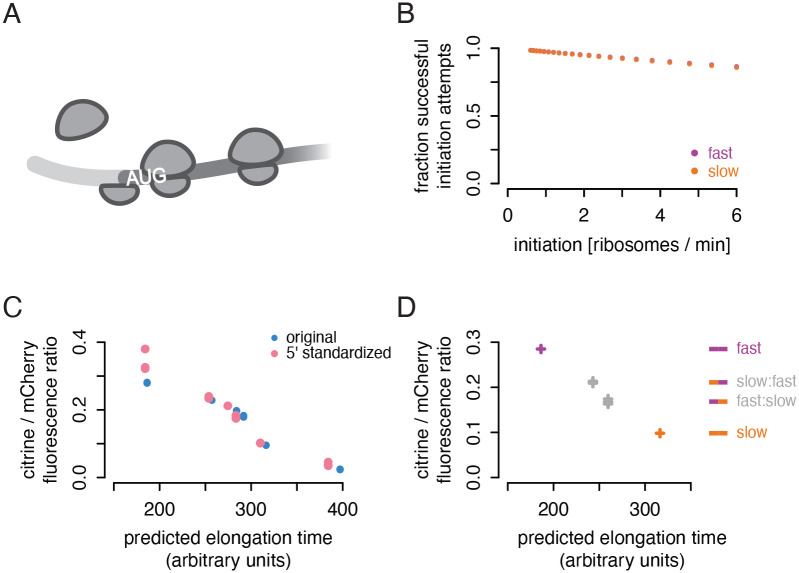
Passive start codon occlusion does not explain lower output of non-optimal sequences. a) Schematic of an initiation interference event; an upstream ribosome cannot initiate because the start codon is occluded by a downstream ribosome. b) Percentage of initiation events that succeed without interference over a range of initiation rates in TASEP simulations. The impact of interference does not differ substantially between fast and slow reporters. c) Flow cytometry measurements of fluorescence from 5′-standardized and original synonymous citrine reporters, as in figure 1B. The first 20 codons of each reporter were standardized to the original yeCitrine sequence. d) Flow cytometry measurements of fluorescence from chimeric, fast, and slow synonymous reporters, as in figure 1B, from three isolates of each chimera and one of each original reporter. The reporters comprise the first half of the slow citrine sequence and the second half of the fast citrine sequence and vice versa.

In some cases, translation initiation can be limited by physical occlusion of the start codon by a ‘traffic jam’ of slowly elongating ribosomes (Plotkin and Kudla 2011; Shah et al. 2013; Chu et al. 2014; Tuller et al. 2010; Riba et al. 2019; Verma et al. 2019). A ribosome covers a footprint of around 30 nucleotides; any ribosome decoding the first ten codons of a transcript would block access to the start codon and interfere directly with initiation (Fig. 4A). This simple passive mechanism, in which slowly elongating ribosomes directly interfere with initiation, could potentially explain the lower protein output of our non-optimal reporters. Analyzing our TASEP simulation, we observed that the start codons on the slow reporter would be occluded only marginally more often than start codons on the fast reporter. At most, occlusion led to an 1% reduction in protein from the slow reporter compared to the fast reporter, when initiation was modeled to be particularly fast (Fig. 4B). In contrast, our experiments showed a 50% reduction in translation efficiency of the slow reporter relative to the fast reporter. The TASEP results indicate that passive traffic jams were unlikely to explain the effect of slow codons on protein output.

To test experimentally whether the difference in output could be explained by the choice of codons proximal to the start codon, we created new versions of each of our six synonymous reporters in which the first 20 codons were standardized to the yECitrine sequence. We scored these new versions with our neural network model, which does not consider the position of a codon in a transcript; it essentially sums the decoding times of all positions in the transcript, with consideration of the impact of adjacent codons. Thus, its prediction of the total protein output of each 5′-standardized reporter only increased by a small percent relative to the original reporter. If codons near the start codon played a central role in limiting initiation, the experimentally measured output would diverge substantially from the neural network prediction; the 5′-standardized sequence would cause fewer traffic jams on the slower reporters and a concomitant increase in protein output. Instead, we observed only a small increase in the ratio of citrine to mCherry fluorescence for all synonymous reporter variants (Fig. 4C). This increase tracked with the slight improvement in predicted elongation time; the 5′-standardized reporters fit the previous trend between total predicted elongation time and protein output. This demonstrates that the protein output of our synonymous reporters is not determined by their 5′ sequence and argues against a 5′ ribosome traffic jam interfering with initiation and limiting output.

### Position of non-optimal codons does not influence protein output

We next asked more generally whether the position of non-optimal codons affected protein output. We recombined opposite halves of our fast and slow citrine reporters to generate slow:fast and fast:slow chimeric variants. We reasoned that if non-optimal codon position were relevant, the chimeric reporters would not track the pre-existing trend between predicted elongation time and protein output, as the elongation time prediction does not take codon position into account. Instead, we observe that the ratio of citrine to mCherry fluorescence closely tracked each chimeric citrine variant’s predicted elongation time (Fig. 4D). The 5′’-non-optimal slow:fast variant displayed slightly higher output than the 5′-optimal fast:slow variant, due to an overall higher fraction of fast codons in the former. This result indicates that there is no strong positional effect of non-optimal codons within these coding sequences.

Taken together, these findings suggest that physical occlusion of the start codon by ribosome traffic jams is not involved in reducing protein output from our non-optimally encoded synonymous reporters. Given that mRNA abundance does not fully explain synonymous reporter output, and that ribosome removal, protein instability, and start-codon-occluding traffic jams are unlikely, we considered that an uncharacterized active mechanism may be reducing initiation on our non-optimal reporters.

### A CRISPRi screen identifies initiation factors as modulators of non-optimal codon translation

**Figure 5:**
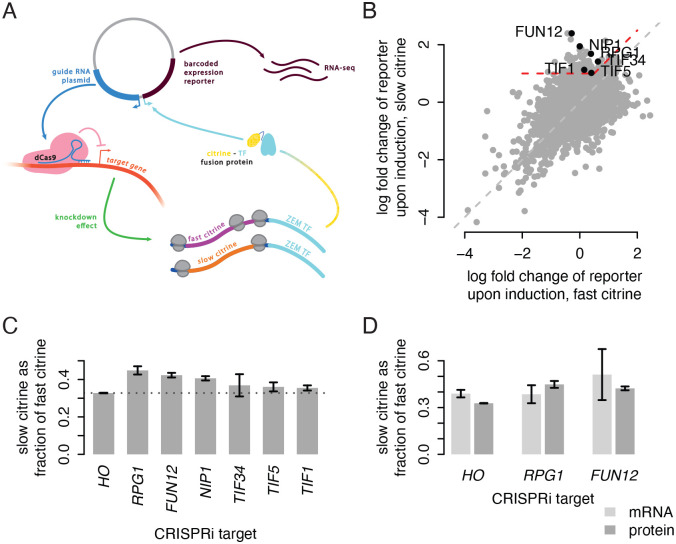
A CRISPRi screen identifies initiation factors as modulators of non-optimal codon translation. a) The modified CiBER-seq assay. Each Cas9 guide RNA cassette is linked to a transcribed reporter with a unique barcode sequence on the same plasmid. Expression of this barcoded reporter is driven by the synthetic transcription factor ZIF286 (ZEM-TF) fused downstream of either the fast or slow synonymous citrine sequence. The RNA-to-DNA ratio for each barcode, determined by deep sequencing, corresponds to reporter expression driven by the citrine-ZEM fusion. A change in translation of the citrine sequence due to CRISPRi knockdown of a relevant gene will result in changes in citrine-ZEM abundance and expression of the barcode linked to that guide RNA. b) Log_2_ fold change (LFC) of barcode counts after guide induction for fast citrine (x-axis) and slow citrine (y-axis) fusion backgrounds, for each linked guide. Red dashed line indicates the region containing guides with linked barcodes that showed LFC > 1 in the slow citrine fusion background and (LFC_slow_ - LFC_fast_) > 0.5. Labels indicate guides that correspond to the term ‘cytoplasmic translation initiation,’ the only overrepresented term in a Gene Ontology analysis of all guides in the region of interest. c) Ratio of normalized fluorescence of the slow citrine reporter to normalized fluorescence of the fast citrine reporter in isogenic CRISPRi backgrounds. Dashed line indicates the value of this ratio for the HO guide background, which is used as a control. Fluorescence was determined by flow cytometry; bar height corresponds to the average of three isolates of the citrine reporter in each guide background and error bars depict standard error of the mean. d) Ratio of slow citrine to fast citrine mRNA abundance (light gray) and fluorescence (dark gray) from HO, RPG1, and FUN12 guide backgrounds. mRNA abundance was determined by RT-qPCR from three isolates, normalized to mCherry mRNA, and fluorescence data are repeated from panel C.

To identify potential mechanisms operating on our reporters, we sought to identify unknown genetic interactions that may modulate translation differently on our fast and slow citrine variants. We modified a pooled, genome-wide CRISPRi screen, CiBER-seq (Muller et al. 2020), by fusing a citrine variant sequence to a synthetic transcription factor that drives expression of a transcription reporter (Fig. 5A; Fig. S3A). Changes in translation of the citrine sequence would change the abundance of the transcription factor fusion and in turn change the expression of the transcription reporter. A pool of plasmids, each containing a unique barcoded reporter and a Cas9 guide RNA against the promoter region of one yeast gene, are transformed into a population of yeast, allowing unique identification of the CRISPRi target in the cell expressing a particular barcode. By sequencing the reporter mRNAs across all cells and counting the frequency of each barcode, we can measure how knockdown of each gene influences citrine translation with greater sensitivity and precision than a standard approach of sorting yeast by fluorescence output.

To find genes with differential effects on fast and slow citrine translation, we transformed the plasmid library into yeast containing either the fast or slow citrine-transcription factor fusion and compared outcomes (Fig. 5B). To identify genes whose inhibition allowed for higher relative expression of slow citrine without a substantial effect on fast citrine, we focused on the 118 guides with a two-fold increase in reporter count in the slow-codon citrine strain and a large difference between the fast and slow citrine strains (Δ(log_2_ fold change) > 0.5). We reasoned that genes displaying this effect upon knockdown would contribute to translational repression of non-optimally encoded sequences. Notably, no guides against known translational quality control genes showed a large difference in effect between the fast and slow citrine backgrounds. Instead, the data revealed an impact of translation initiation. We found two significantly overrepresented Gene Ontology terms within this set: ‘cytoplasmic translation initiation’ (p = 0.03, hypergeometric test with Benjamini-Hochberg false discovery rate < 0.05) and the more specific ‘formation of cytoplasmic translation initiation complex’ (p = 0.002). The first term was represented by six initiation factor genes: *RPG1*, *NIP1*, and *TIF34* (eIF3 subunits a, c, and i respectively); *FUN12* (eIF5B); *TIF1* (eIF4A); and *TIF5* (eIF5). The second term included all these genes except eIF4A. The presence of three subunits of eIF3 could suggest a specific role for this complex, and we note that a guide against the g subunit *TIF35* also showed a strong differential effect below our threshold.

### Initiation factor depletion reduces difference in synonymous reporter output

We verified the results of the CiBER-seq screen by measuring the effect of initiation factor knockdown on citrine translation more directly using flow cytometry. We created individual yeast strains with either the fast or slow citrine reporter, CRISPRi machinery, and inducible guides against each of the six initiation factors and against the HO locus as a control for general effects of CRISPRi. To confirm that our results were not a general result of growth defects, we also tested two guides that would cause large growth defects but showed no differential effect in the CiBER-seq screen (Fig. S3B). We compared the ratio of slow to fast citrine fluorescence between the initiation factor targets and the HO control and found that knocking down all targets except *TIF34* showed an increase in the ratio (Fig. 5C). We conclude that, while translation of all genes likely decreases when initiation factors are depleted, this decrease is less pronounced for our slow reporter. Thus, the impact of non-optimal codons on translation is lessened when initiation factors are disrupted.

To determine if initiation factor depletion affected translation efficiency or if it mainly acted via a change in translation-dependent mRNA degradation, we measured citrine and mCherry mRNA after CRISPRi depletion of eIF3a and eIF5B, the two initiation factors with the largest effect in our screen (Fig. 5D). We observed that the ratio of slow to fast mRNA after eIF3a depletion was unchanged compared to the HO control, while the fluorescence ratio increased. This indicates that eIF3a has a differential effect on the number of proteins produced per mRNA from the two reporters. The mRNA abundance measurements after eIF5B depletion were too variable to be conclusive but pointed to stabilization of mRNA from the slow reporter rather than increased translation efficiency.

Translation initiation is clearly implicated, and our next goal was to distinguish between two possibilities. The effect could be a straightforward consequence of having fewer translating ribosomes after initiation factor depletion. Or, our knockdowns could have disrupted a specific active mechanism that regulates non-optimal codon translation, perhaps dependent on eIF3. This led us to wonder if disrupting initiation in an orthogonal manner would result in a similar change in the relative translation efficiency of our synonymous reporters.

### Lowering initiation with 5′ UTR stem-loops attenuates the difference in protein output

**Figure 6:**
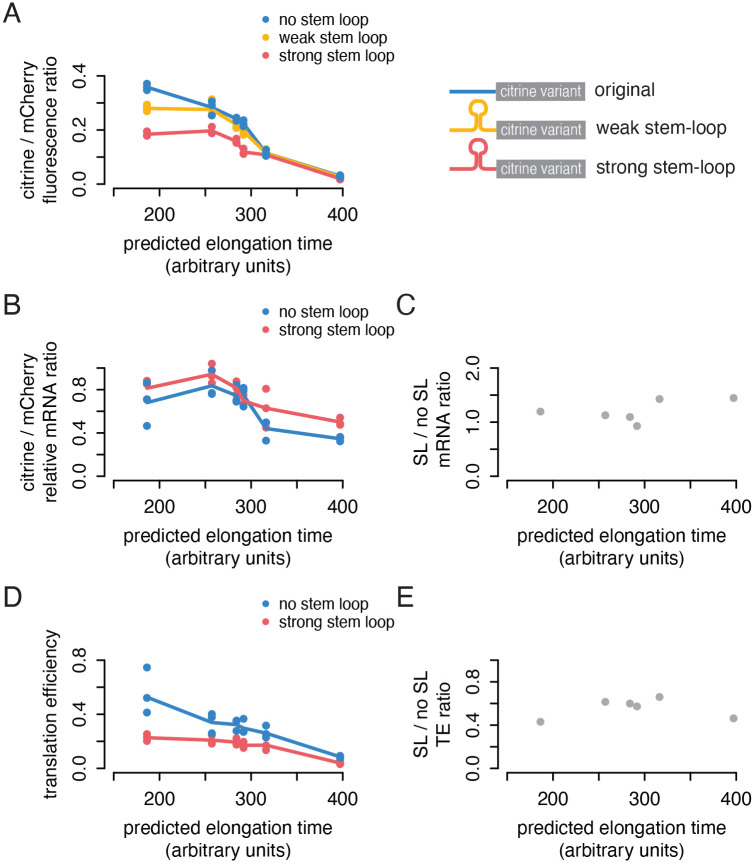
The impact of non-optimal codons on protein output, but not translation efficiency, depends on initiation rate. a) Flow cytometry measurements of fluorescence from synonymous citrine reporters with and without stem-loop structures in the 5′ UTR, as in figure 1B. Lines represent the average of three replicates.b) mRNA abundance of strong stem-loop and original synonymous reporters normalized to mCherry mRNA abundance, determined by RT-qPCR. c) Relative change in mRNA abundance caused by strong stem-loop for each reporter, shown as the ratio of stem-loop (SL) reporter mRNA to original reporter mRNA. d) Translation efficiency, shown as fluorescence divided by mRNA abundance, of the strong stem-loop and original synonymous reporters. e) Relative change in translation efficiency (TE) caused by strong stem-loop for each reporter, shown as the ratio of stem-loop (SL) reporter TE to original reporter TE.

To further understand how lowering translation initiation affects the difference in protein output from our reporters, we inserted RNA stem-loop (SL) structures with different folding energies, strong-SL and weak-SL, directly upstream of the start codon in each citrine construct. These stem-loop structures have been shown to reduce protein expression in proportion to their folding energy and are thought to function by interfering with 48S preinitiation complex (PIC) scanning (Weenink et al. 2018; Wang et al. 2022). We measured the fluorescence of these SL-reporter variants via flow cytometry, normalizing to the mCherry reporter (Fig. 6A). We observed that the stem-loops had a varied, non-linear effect across our reporters. The weak stem-loop lowered the output of only the fastest reporter, while the strong stem-loop lowered the output of all but the two slowest reporters. Overall, we see a shift from initiation-limited to elongation-limited translation as elongation speed becomes slower or initiation becomes faster.

To separate effects of the stem-loop on mRNA degradation from effects on translation efficiency of the reporters, we measured the abundance of citrine mRNA in the six strong-SL reporter strains and the six original reporter strains, normalized to mCherry mRNA (Fig. 6B). Citrine mRNA abundance was higher for all stem-loop reporters compared to their non-stem-loop counterparts. The slower reporters were slightly more stabilized than the fast reporters (Fig. 6C), suggesting that their stabilization depends in part on elongation speed and codon content. This aligns with the idea that the degree of co-translational decay depends on initiation rate, as less initiation results in fewer slow ribosomes to trigger decay.

### Lower translation efficiency of non-optimal reporters is maintained across initiation rates

To see if the differential increase in mRNA abundance explained the non-linear effect of the stem-loop on synonymous reporter protein output, we calculated the translation efficiency of our reporters as the ratio of citrine fluorescence to mRNA abundance (Fig. 6D). The translation efficiency of the stem-loop reporters scales nearly linearly with codon optimality, just as we observed for the original reporters, albeit over a smaller range due to overall lower protein output with the stem-loop. The stem-loop consistently reduced the translation efficiency by around half relative to each non-stem-loop reporter (Fig. 6E). In all, the pattern of protein output for the stem-loop reporters results from a combination of decreased translation efficiency and increased mRNA stability.

Importantly, the stem-loop reduces the translation efficiency of all reporters by a similar degree regardless of predicted elongation time. Even in the context of slower initiation, slower coding sequences produce fewer proteins per mRNA in a given time than faster coding sequences, in a remarkably linear way. We infer that some mechanism must detect slow elongation and trigger production of fewer proteins per mRNA, most plausibly by lowering initiation, and this mechanism is not disrupted by stem-loops that also lower initiation. The observation that eIF3a depletion alters relative protein output without changing reporter mRNA ratios and the identification of multiple subunits of eIF3 in the genome-wide screen may indicate a specific role for eIF3 in mediating the impact of non-optimal codons on translation by lowering translation initiation.

## Discussion

In this study, we have established that codon choice and translation elongation speed can directly determine translation efficiency and protein output across a wide range of codon optimality, and that this effect must involve unexpected mechanisms. Our observations highlight the variable balance between initiation rate, elongation speed, and mRNA turnover in determining protein output, and point to a mechanism actively responding to slow elongation to decrease initiation on non-optimal transcripts.

We have investigated and ruled out mechanisms that are known to link slow elongation speed to reduced expression. We see that the well-established link between slow elongation and mRNA turnover does have a major impact on our reporters, but that this explains less than half of the difference in protein output. Looking beyond that mechanism, we tested known translational quality control mechanisms but found that none were sufficient to explain the observed difference in output between our synonymous reporters, which may not cause the extreme stalls and collisions that trigger quality control (Veltri et al. 2022; Hou et al. 2023).

We then systematically investigated the hypothetical ways that protein output from an mRNA might be limited: by causing fewer ribosomes to start translation, causing fewer ribosomes to successfully complete translation, or creating defective or unstable proteins. We ruled out the possibilities of incomplete translation and protein instability by examining the expression of fluorescent reporters separated by a skipping element. The fluorescence ratio between the two reporters was unaffected by the codon content of the downstream reporter. We also ruled out an effect of passive ‘traffic jams’ occluding the start codon of the slower reporters.

Collectively, these observations lead us to propose that slow elongation limits translation initiation through an unknown, active mechanism. This model is corroborated by a recent report identifying depletion of cap binding proteins from non-optimal reporter transcripts; our results align with the observations of lower initiation in that study (Barrington et al. 2022). The discovery of specific feedback on initiation in response to ribosome stalls (Hickey et al. 2020; Juszkiewicz et al. 2020; Sinha et al. 2020) makes it plausible that an analogous mechanism could function on slow but non-stalled ribosomes. How this process might occur on non-optimal but functional genes motivated our CRISPRi screen.

The results of our genome-wide CRISPRi screen emphasize the interplay between translation initiation and elongation and hint at possible feedback mechanisms. Translation initiation factors, including multiple subunits of eIF3, were enriched among the genes whose depletion reduced the difference in output between our fast and slow reporters. We found a similar effect on protein output from lowering initiation by inserting a stem-loop structure in the 5′ UTR of our reporters. While these two experiments had a common focus on initiation, they may suggest two distinct processes at play. Reducing initiation with a stem-loop may protect slowly translated mRNAs from translation-dependent decay by reducing the total number of slow ribosomes that trigger this process. This aligns with evidence that endogenous genes have evolved 5′ UTRs that set their translation initiation to be concordant with their codon optimality (Park and Subramaniam 2019; Riba et al. 2019). However, beyond the direct effect of lowering initiation, there is still a difference in translation efficiency between fast and slow stem-loop reporters. Depletion of eIF3a caused a distinct change in translation efficiency without affecting mRNA levels, potentially indicating that the eIF3 complex is directly involved in setting the translation efficiency of non-optimal sequences. Many studies have pointed to diverse roles for eIF3 across all stages of translation, including initiation (Martineau et al. 2008), elongation (Pöyry, Stoneley, and Willis 2020; Lin et al. 2020; Wagner et al. 2020; Bohlen et al. 2020) and termination (Beznosková et al. 2013), and its impact on initiation may go beyond its canonical role as a scaffold for pre-initiation complex formation.

## Methods

### Plasmid and yeast strain construction

The citrine variant sequences used and modified in this study were previously described in (Tunney et al. 2018). All fluorescent protein expression was directed by a PGK1 promoter and an ADH1 terminator.

Citrine variants were integrated into the yeast genome using the plasmids described in (Tunney et al. 2018), containing a *K. lactis* LEU2 expression cassette and two 300bp sequences homologous to the *his3Δ1* locus of BY4742 for homologous recombination. pPGK1-mCherry was amplified from the mCherry plasmid described in (Tunney et al. 2018) using uracil-containing primers with Q5U polymerase (New England BioLabs, M0515) and inserted into EasyClone plasmid pCfB2226 by USER cloning upstream of the ADH1 terminator with USER Enzyme Mix (NEB, M5508) according to (Stovicek et al. 2015). The resulting mCherry plasmid contains an *S. pombe* his3 expression cassette for selection and integrates into the *S. cerevisiae* X-4 chromosomal locus.

All subsequent amplifications for cloning purposes used Q5 2X MasterMix (NEB, M0492). Knockout constructs were created by amplifying either a KanMX or HygR resistance marker from a parent plasmid with two sets of overlapping 60nt primer pairs, generating amplicons consisting of the selectable marker flanked by 80–100bp of target locus homology.

Dual fluorescence E2A reporter constructs were constructed as follows. The E2A sequence was divided into halves that overlapped by 15nt. Fast and slow citrine plasmids were linearized by amplification. The forward primer for each began at the citrine start codon and was tagged with the downstream half of the E2A sequence. The reverse primer began directly upstream of the start codon. Amplified plasmids were digested with DpnI (NEB, R0176). The mCherry coding sequence was amplified with a forward primer located at the mCherry start codon that was tagged with citrine vector homology. The reverse primer began one codon before the mCherry stop codon and was tagged with the upstream half of the E2A sequence. The mCherry insert was assembled into the citrine vector using NEB HiFi DNA assembly (NEB, E2621). The emiRFP reporter was constructed by linearizing the mCherry plasmid backbone with primers positioned to exclude the mCherry coding sequence. The emiRFP was amplified from a donor plasmid with primers containing mCherry backbone homology. The emiRFP insert was assembled into the mCherry backbone using NEB HiFi DNA assembly.

5′-standardized citrine variant plasmids were constructed by whole plasmid PCR of each variant using opposing primers containing the standardized codon sequence. Amplified plasmid was gel purified and then circularized with KLD Enzyme Mix (NEB, M0554).

Chimeric citrine reporter plasmids were constructed by amplifying each half of the fast and slow citrine sequences. A citrine plasmid was double digested at the *PacI* and *AscI* site to remove the citrine coding sequence from the backbone and treated with Quick CIP (NEB, M0525) to prevent recircularization. The up- and downstream halves of each chimeric citrine variant were assembled into the backbone using HiFi DNA Assembly MasterMix with a 60nt ssDNA oligo bridge according to manufacturer’s instructions.

Individual CRISPRi guide expression plasmids were constructed by inserting a tet-inducible guide expression cassette from (Muller et al. 2020) into EasyClone plasmid pCfB2188, containing the KanMX marker for selection, via USER cloning according to (Stovicek et al. 2015). Individual guide sequences were ordered as 60nt ssDNA oligos containing the 20nt guide sequence flanked by 20nt backbone homology on either side, and were integrated into the *AvrII* site using HiFi DNA Assembly MasterMix.

Stem-loop citrine reporter plasmids were constructed by assembling four overlapping 60nt ssDNA oligos containing the stem-loop sequence into the *PacI* site of each citrine variant using HiFi DNA Assembly MasterMix according to manufacturer’s instructions.

All USER reaction products and HiFi assembly products were transformed into chemically competent *E. coli* cells (NEB 5-alpha: C2987, Agilent through UCB MacroLab XL1-Blue: 200249) according to the manufacturer’s directions. Plasmids were harvested from overnight cultures using either Zymo Miniprep Kits (Zymo, 11–308) or NEB Miniprep Kits (NEB, T1010).

All plasmids were sequence confirmed and transformed into yeast using the high-efficiency lithium acetate/single-stranded carrier DNA/PEG method according to (Gietz and Woods 2002). Citrine plasmids were linearized at the *SbfI* site, and ~1 *μ*g linearized plasmid was used to transform BY4742 yeast. mCherry plasmid was linearized at the *NotI* site, and ~1ug linearized plasmid was transformed into BY4741 yeast. Citrine transformants were selected by growth on SCD-Leu plates and mCherry transformants were selected on SCD-His plates. Knockout constructs were transformed into mCherry and citrine yeast backgrounds, which were then grown on YEPD plates overnight, then replica plated onto the appropriate antibiotic plates for selection. CRISPRi strains were constructed by transforming the dCas9-Mxi expression plasmid from (Muller et al. 2020) into the mCherry yeast background and selected for on kanamycin plates. CRISPRi guide expression plasmids were linearized at the *NotI* site, and ~1ug of linearized plasmid was transformed into the mCherry yeast background and selected for on SCD-Ura plates.

Transformant colonies were picked from selective plates and cultured overnight at 34°C in 1mL selective liquid media in deep-well 24-well blocks supplemented with one glass bead per well, shaking at 750rpm in a Multitron incubator shaker. Genomic integration of all constructs was confirmed by using 1uL of liquid culture in a colony PCR reaction with Phire Green Hot Start II PCR Master Mix (ThermoFisher, F126) with primers flanking the integration site. Genotyped transformant cultures were struck out onto selective plates and preserved as glycerol stocks in 30% glycerol at −80°C.

Diploid strains expressing both citrine and mCherry were created by inoculating 1mL of YEPD with one BY4741 strain and one BY4742 strain, which was then incubated overnight in 24-well blocks as described above. Overnight mating cultures were then struck out onto SCD-Met-Lys plates for diploid selection. To create diploid knockout strains, three isolates of each haploid mCherry knockout strain were mated with separate isolates of each haploid citrine knockout strain, yielding a total of three diploid biological replicates with no parental isolate backgrounds in common. All other diploid yeast strains were created by mating one haploid isolate of mCherry yeast to three separate isolates of haploid citrine yeast, yielding three biological replicates that share the same parental mCherry isolate background.

### Yeast culture

Yeast strains were cultured overnight in either YEPD or selective dropout media, then back-diluted into YEPD to an OD_600_ of 0.1 and shaken at 250 rpm at 30°C in a sterile 24-well deep-well block supplemented with a sterile glass bead until their OD_600_ was between 0.4 and 0.7.

CRISPRi yeast strains were cultured overnight in SCD-Met-Lys, then back diluted into either SCD or YEPD to an OD_600_ of 0.1, and shaken at 250 rpm at 30°C in a sterile 24-well deep-well block supplemented with a sterile glass bead until their OD_600_ was between 0.4 and 0.7. Cultures were then serially back diluted into SCD or YEPD with tetracycline at final concentration of 250ng/uL, to an OD_600_ of 0.0005, 0.0001, and 0.00005, then grown overnight by the same method as above. Serial dilutions were necessary because CRISPRi strains have differing degrees of growth defect. CRISPRi-induced yeast were collected after at least 9 doublings at an OD_600_ between 0.4 and 0.7.

### Flow cytometry and analysis

200uL aliquots of suspended mid-log phase culture was pelleted by centrifugation at 3000*g* for 5 minutes in a 96-well U-bottom plate, washed with 200uL of DPBS (Gibco, 14190–44), fixed in 4% formaldehyde for 15 minutes in the dark at room temperature, then washed twice and diluted 1:4 in DPBS and stored at 4°C.

Flow cytometry of fixed yeast was carried out on a BD Biosciences LSR Fortessa analyzer using a High Throughput Sampler. Forward light-scatter measurements (FSC) for relative size and side-scatter measurements (SSC) for intracellular refractive index were made with a 488-nm laser. Citrine fluorescence was measured with 488-nm (blue) laser excitation and detected with a 505-nm long-pass optical filter followed by a 525/50 nm optical filter with a bandwidth of 50 nm. mCherry fluorescence was measured with a 561-nm (yellow–green) laser for excitation and a 600-nm long-pass optical filter followed by 610/20-nm band-pass optical filter with a bandwidth of 20 nm. emiRFP fluorescence was measured with a 561-nm (yellow–green) laser for excitation and a 635-nm long-pass optical filter followed by 670/30-nm band-pass optical filter with a bandwidth of 30 nm. PMT values for each color channel were adjusted such that the mean of a sample of BY4743 yeast was 100. 50,000 events were collected for each biological sample.

Flow cytometry data were analyzed with a custom R script (available on Github in “analysis code” folder) whose core functionality is based on the Bioconductor packages flowCore, flowStats, and flowViz. To select events that represented normal cells, we used the norm2filter method to extract events that had FSC and SSC values within the region of highest local density of all events. This gating method fits a 2D normal distribution and selects all events within the Mahalanobis distance. For these gated events, the red and yellow fluorescence intensities were normalized by subtracting median red and yellow intensities from a control sample with no fluorescent reporters grown in the same media and conditions. For each sample, we computed the citrine to mCherry ratio as the median ratio of the background-corrected yellow and red intensities.

### CiBER-seq assay

#### Strain and plasmid construction and turbidostat culture

The inducible gRNA-barcode plasmid library, the citrine-ZIF286 fusion protein plasmid, and the dCas9-Mxi1-TetR plasmid used in the modified CiBER-seq assay were generated as described in (Muller et al. 2020). Yeast strains containing either a fast or slow citrine-ZIF286 fusion and the dCas9 plasmid were generated according to the yeast transformation protocol described above. These strains were then transformed separately with aliquots of the same pooled gRNA-barcode plasmid library, and serial dilutions were plated to ensure sufficient transformation efficiency was achieved. In all subsequent steps SCD-Ura media was used to maintain the non-integrating guide plasmid. Transformants were shaken overnight at 250 rpm in a 30° shaking water bath, then back diluted to an OD_600_ of 0.1, and allowed to grow until mid log phase. A custom made turbidostat (McGeachy, Meacham, and Ingolia 2019) was then inoculated with both fast and slow CiBER-seq yeast strains in two replicate growth chambers, and allowed to run continuously until an OD_600_ equal to 0.8 was reached. Media used in the turbidostat was supplemented with 8 nM beta-estradiol to allow ZIF286 nuclear import. When the growth rate reached steady state (90 minute doubling time), 50mL pre-induction samples were collected. gRNA expression was then induced by adding anhydrotetracycline in DMSO into both of the growth chambers and the media reservoir to a final concentration of 250 ng/ml. 50mL post-induction samples were collected six doublings later, after approximately 9 hours. Collected samples were centrifuged at 4000 × g for 5 min, the media was aspirated, and the pellets were stored at −80°C.

#### DNA and RNA library preparation

Plasmid DNA was extracted from yeast using Qiagen QIAprep Spin Miniprep Kits (Qiagen, 27104). 500uL Resuspension Buffer PI was added to thawed pellets, then 100*μ*L acid-washed glass beads (Sigma, G-8772), followed by vortexing for 10 minutes. 500*μ*L Lysis Buffer P2 was added, and tubes were gently inverted 4–6 times, then incubated at room temperature for 5 minutes. 700*μ*L Neutralization Buffer N3 was added, and tubes were inverted 4–6 times, then centrifuged at maximum speed for 10 minutes. Cleared lysate was transferred to a spin column, flowed through, washed with 750*μ*L Wash Buffer PE, and spun dry. DNA was eluted by adding 45uL pre-heated nuclease-free water (37°C), then incubating for 5 minutes at room temperature before centrifugation. Eluate was digested with *PvuII-HF* (NEB, R3151S) for 2–3 hours, then column cleaned in a Zymo 5*μ*g DNA Clean and Concentrate column (Zymo, D4004). In vitro transcription was performed using a T7 Quick HiScribe kit (NEB, E2050S) according to manufacturer’s instructions with overnight incubation at 37°C, then treated with DNase. IVT product was then reverse transcribed with a ProtoScript II kit (NEB, E6560S) according to manufacturer’s instructions, then treated with RNase A and RNase H. Purified product was amplified with distinct NEBNext dual index primer sets (NEB, E7600S) for 6 cycles with Q5 polymerase in 50uL reactions.

RNA was isolated from yeast using acid phenol chloroform extraction according to (Ares 2012), then reverse transcribed using ProtoScript II with oligo-dT primers according to manufacturer’s instructions. cDNA product was treated with RNase H and RNase A, purified in a Zymo 5*μ*g column, and amplified for 6 cycles with Q5 polymerase using primers RM512 and RM546 from (Muller et al. 2020). This reaction was purified and amplified with distinct NEBNext dual index primer sets as above.

#### Computational analysis

RNA and DNA barcode reads were trimmed with cutadapt and quantified with custom scripts available in the Github repository. Barcodes with at least 24 reads in each DNA library were kept for analysis. Pre- vs post-induction RNA and DNA barcode abundance in two replicate turbidostat runs were analyzed with the ‘mrpa’ R package (Myint et al. 2019) to compute log fold changes of each barcode upon induction for the fast and slow reporter experiments.

### mRNA quantification by qRT-PCR

All qRT-PCR samples were prepared from frozen pellets from 1mL mid-log phase yeast culture. Total RNA was extracted using one-step hot formamide extraction, according to (Shedlovskiy, Shcherbik, and Pestov 2017). In brief, 25uL of a solution of 98% formamide and 10mM EDTA was used to resuspend 0.5 OD_600_ units of yeast and heated at 70°C for 10 min before being centrifuged at 21x *g* for 2 minutes. Supernatant containing the RNA was collected. RNA concentration was measured on a NanoDrop and 50*μ*g RNA was purified using an RNA Clean & Concentrator-25 Kit (Zymo, R1017), with on-column DNaseI digestion, as per manufacturer’s instructions. RNA was eluted in 50*μ*L of nuclease-free water and measured on a NanoDrop. 1*μ*g of RNA was reverse transcribed with oligo(dT)_16_ primers and SuperScript IV (Invitrogen, 18090010) according to the manufacturer’s instructions. One-tenth of this reaction (2*μ*L) was then subjected to qPCR with a DyNAmo HS SYBR Green qPCR Kit (Thermo Scientific, F410L) according to manufacturer instructions on a CFX96 Touch Real Time qPCR Detection System (Bio-Rad, 1845096 with 1851196). For each RT reaction, two sets of qPCR reactions were performed; one with primers specific to mCherry and one with primers specific to the citrine variant being probed, with each set consisting of three technical replicates. qPCR data were analyzed with the ΔΔCq method using custom R scripts (available on Github). The signal from each citrine variant was normalized to mCherry signal from the same biological sample.

### Polysome profiles

#### Yeast vacuum filtration and cryogrinding

Overnight cultures of slow and fast citrine yeast were back-diluted so that each strain was at an OD of 0.05, for a combined OD of 0.1 in 125mL YEPD. Two mixed cultures were prepared, each consisting of different biological replicates of each citrine strain. Mixed cultures were shaken at 300 rpm at 30°C until they reached mid-log phase, at which point they were vacuum filtered using a sintered glass filter and 0.45 uM nitrocellulose membranes, then flash frozen in a falcon tube filled with LN2.

LN2 level was adjusted so that the yeast pellet was covered, then 2 mL of lysis buffer (10 mM Tris pH 7.0, 10 mM Tris pH 8.0, 150 mM NaCl, 5mM MgCl2, 1 mM DTT, 0.1mg/mL Cycloheximide, 0.01% Triton-X, 0.0024 U/uL Turbo DNase) was added dropwise into sample. After washing, drying, and cooling the chamber and ball bearing of the Mixer Mill 400 (Retsch, 20.715.0001) in LN2, frozen yeast and lysis buffer was added to the chambers and shaken at 15 1/s frequency for 3 minute intervals 5 times. This cryoground lysate was then thawed, clarified via centrifugation, and its total RNA concentration was quantified using a Quant-It kit (ThermoFisher, Q33140).

#### Polysome fractionation and RNA extraction

To separate the polysomes, a standard polysome fractionation procedure was followed. Briefly, 10% sucrose (10% Sucrose, 10 mM Tris pH 7.0, 10 mM Tris pH 8.0, 150 mM NaCl, 5mM MgCl2, 0.1mg/mL Cycloheximide, 0.002 U/uL SuperAseIN) and 50% sucrose (50% Sucrose, 10 mM Tris pH 7.0, 10 mM Tris pH 8.0, 150 mM NaCl, 5mM MgCl2, 0.1mg/mL Cycloheximide, 0.002 U/uL SuperAseIN) solutions were prepared, then 8mL of 50% sucrose was layered under 6mL of 10% sucrose in Seton Open-Top Polyclear Centrifuge Tubes (Seton, 7030). Gradients were established using Biocomp Instruments’ Piston Gradient Fractionator (made up of part no.: 153, 105–914A-IR, and F-1–260). 200uL of clarified lysate was layered on top of these gradients and spun in a Beckman ultracentrifuge with a SW41 rotor at 36,000 rpm for 3 hours at 4°C. These gradients were then fractionated using the Piston Gradient Fractionator. Fractions were collected based on the polysome traces generated by the UV monitor, and subsequently flash frozen in LN2.

A constant amount of total RNA isolated from HEK293 (1uL of 500 ng/uL) was then spiked into each fraction and mixed. 500uL of each spiked-in fraction was aliquoted, ethanol precipitated overnight, and treated with DNaseI according to manufacturer’s instructions. Acid phenol chloroform extraction was then performed according to (Ares 2012) to isolate the total RNA of each sample, and samples were ethanol precipitated once more and resuspended in water.

#### RNA-seq library preparation and analysis

These samples were sent to UC Berkeley’s Functional Genomics Laboratory for quantification and quality check via bioanalyzer, Poly-A selection to enrich for mRNA, and Illumina library preparation. Samples were pooled in equimolar ratios, and Illumina sequencing was performed with the NovaSeq 6000 in an SP flow cell, with 100 single-read sequencing cycles.

To quantify citrine mRNAs in each fraction, RNA-seq reads were aligned with bowtie2 to a custom yeast transcriptome file containing all yeast genes plus the six citrine sequences. To calculate normalization from human mRNA spike-ins, reads were also aligned to the human transcriptome (allowing one alignment per read to avoid double-counting reads). The number of reads matching the fast and slow reporter in each polysome fraction was divided by the total number of human reads in that fraction. Analysis code is available on github.

### Translation simulations

We carried out stochastic simulations of translation that modeled the translation of an mRNA, followed by its eventual degradation, using a totally asymmetric simple exclusion process (TASEP) (MacDonald and Gibbs 1969; Zia, Dong, and Schmittmann 2011). This simulation tracked the positions of ribosomes on a model transcript using the following elementary reactions:

- ***Ribosome loading:*** the recruitment of a small ribosomal subunit and scanning for the start codon. Loading is blocked when the mRNA is already occupied by another pre-initiation complex in the joining state (see below), or by another ribosome within 10 codons of the start codon.
- ***Subunit joining:*** the joining of a large and a small ribosomal subunit at the start codon. Joining was modeled with 3 independent sub-steps; upon completion of the final joining substep, the ribosome transitioned into an elongating state on the first codon.
- ***Elongation:*** translocation of a ribosome one codon along the mRNA. Elongation is blocked by another ribosome within 10 codons of the destination codon. Elongation on the stop codon represents translation termination, which immediately removes the ribosome and releases a completed protein.
- ***mRNA degradation:*** the transcript is instantaneously degraded and no protein is produced by ribosomes engaged in elongation.

We simulated initiation rates between 0.1 and 0.01 s^−1^, corresponding to an average interval of 10 to 100 seconds between initiation events. We chose 21 different values for the overall initiation rate, spaced logarithmically across this interval. The ribosome loading rate was set to twice the overall initiation rate, and the rate of each sub-step of the joining process was set to six times the overall initiation rate.

We determined per-position elongation rates by multiplying a “base” elongation rate of 6 s^−1^ by a per-position scaling factor determined from the iχnos model. The iχnos model predicts the time the ribosome spends on each position of a coding sequence as a function of the identity of the codon being decoded in the ribosomal A site as well as the adjacent codons in the P and E sites and neighboring sequence. The total elongation time modeled by iχnos for the slow reporter was 1.7 times longer than the total elongation time for the fast reporter.

We used an 0.0011 s^−1^ mRNA degradation rate for the fast reporter, corresponding to a 15 minute lifetime. This value is close to the half-lives measured for *PGK1* and *ADH1* transcripts, which were used to create the 5ʹ and 3ʹ UTRs of the reporters. We accelerated mRNA degradation of the slow reporter 1.54-fold to match the measured steady-state abundance ratio between the slow and fast reporters, under the assumption that their transcription rates were identical.

We carried out 16,384 replicate simulations for each parameter set using the Gillespie algorithm (Gillespie 1976) and recorded mRNA lifespan, protein production, and the frequency of “failed” reactions that were blocked by conflicts with other ribosomes on the same mRNA.

## Supplementary Material

1

## Data Availability

All analysis code and data are available at https://github.com/lareaulab/synonymous-citrines
