# Supplementary material for: Translation elongation as a rate limiting step of protein production
Source: bioRxiv. 2024 Aug 30:2023.11.27.568910. Originally published 2023 Nov 28. Preprint. [Version 2] doi: 10.1101/2023.11.27.568910 (PMC10705293; doi:10.1101/2023.11.27.568910)
Supplement: 1 [file NIHPP2023.11.27.568910V2-supplement-1.pdf]

bioRxiv preprint doi: https://doi.org/10.1101/2023.11.27.568910; this version posted August 30, 2024. The copyright holder for this preprint (which was not certified by peer review) is the author/funder, who has granted bioRxiv a license to display the preprint in perpetuity. It is made available under aCC-BY 4.0 International license.

Supplemental Figures

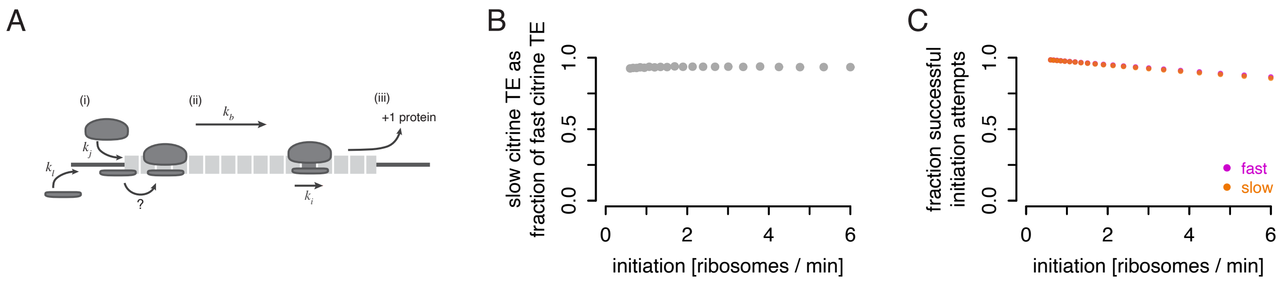

Figure S1 (corresponds to Fig. 2 and 4): Schematic of the basic Totally Asymmetric Simple Exclusion Process (TASEP) model simulating translation of our citrine sequences. There are four steps: (i) pre-initiation complexes will load on to the 5′ end of the mRNA and scan for the start codon with loading rate k_l, as long as they are not blocked by another ribosome overlapping the start codon. (ii) Ribosomal subunits will then join to form an elongation complex with three independent steps, each at rate k_j. (iii) As long as there is no ribosome blocking the one upstream, ribosomes will move to the next codon with rate k_i, which is equal to the base elongation rate, k_b multiplied with the elongation rate factor at every position i. This factor i is relative, and is calculated as (1/ occupancy rates) from the iXnos algorithm. (iv) Ribosomes terminate when they reach the end of the coding sequence, and one protein is counted. b) The difference in the ratio of slow to fast simulated translation efficiency (protein/mRNA*time) from the TASEP model is very small across a range of realistic initiation rates. c) Percentage of initiation events that succeed without interference over a range of initiation rates in TASEP simulations. The impact of interference does not differ substantially between fast and slow reporters.

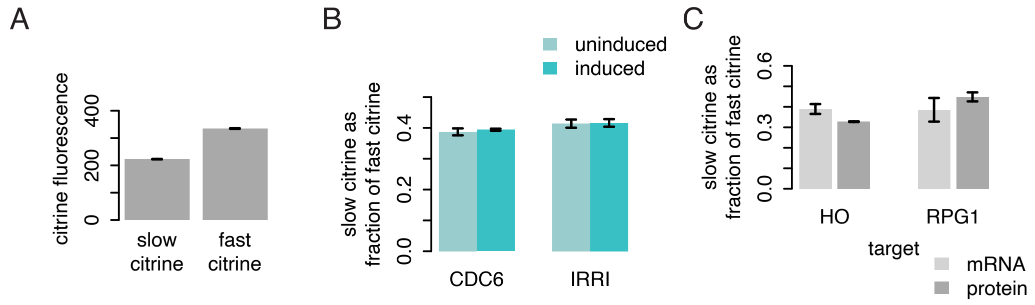

Figure S2 (corresponds to Fig. 5): a) Fluorescence of the ZEM TF–citrine fusions used in the CiBER-seq screen was measured with flow cytometry. The medians of ~15,000 events are plotted after background correction, showing a difference in fluorescence of slow vs fast fusions. b) Ratio of normalized fluorescence of the slow citrine reporter to normalized fluorescence of the fast citrine reporter, as in figure 5C, in strains with CRISPRi guides against genes that are not involved in translation but whose knockdown causes large growth defects (McGlincy et al., 2021). The strains exhibit no differential effect on citrine fluorescence. c) Relative abundance of mRNA from slow vs fast citrine reporters was similar between CRISPRi knockdown of RPG1 and the HO locus control. mRNA abundance (light gray) was determined by RT-qPCR from three isolates, normalized to mCherry mRNA. Data showing a change in fluorescence ratios (dark gray) are repeated from figure 5C for comparison.
